# Taxonomic review of New World Tachyina (Coleoptera, Carabidae): descriptions of new genera, subgenera, and species, with an updated key to the subtribe in the Americas

**DOI:** 10.3897/zookeys.626.10033

**Published:** 2016-10-20

**Authors:** Olivia F. Boyd, Terry L. Erwin

**Affiliations:** 1 Department of Integrative Biology, Oregon State University, Corvallis, OR 97331 USA; 2 Department of Entomology, National Museum of Natural History, Smithsonian Institution, P.O. Box 37012, Washington, D.C. 20013–7012 USA

**Keywords:** Elaphropus, Meotachys, Nototachys, Polyderidius, Polyderis, Nothoderis, Stigmatachys, Tachyxysta, Amazon basin

## Abstract

The classification of the carabid subtribe Tachyina (Trechitae: Bembidiini) is reviewed in light of newly discovered diversity from Central and South America. Described herein are three new genera (*Tachyxysta* **gen. n.**, *Stigmatachys* **gen. n.**, *Nothoderis* **gen. n.**), two new subgenera of *Meotachys* (*Scolistichus* **subgen. n.**, *Hylotachys* **subgen. n.**), and two new subgenera of *Elaphropus* (*Ammotachys* **subgen. n.**, *Idiotachys* **subgen. n.**). Two names previously synonymized under *Polyderis* (*Polyderidius* Jeannel, 1962) and *Elaphropus* (*Nototachys* Alluaud, 1930) are elevated to generic and subgeneric status, respectively. Eight new species are recognized: *Tachyxysta howdenorum* (type locality: México: Chiapas: El Aguacero, 680m); *Elaphropus marchantarius* (type locality: Brazil, Amazonas, Rio Solimões, Ilha de Marchantaria), *Elaphropus acutifrons* (type locality: Brazil: Pará, Santarém) and *Elaphropus occidentalis* (type locality: Perú: Loreto, Pithecia, 74°45'W 05°28'S); *Stigmatachys uvea* (type locality: Perú: Loreto: Campamento San Jacinto, 2°18.75'S, 75°51.77'W, 175–215m); and *Meotachys riparius* (type locality: Colombia: Amazonas: Leticia, 700 ft), *Meotachys ballorum* (type locality: Brazil: Amazonas, Rio Negro Cucui), and *Meotachys rubrum* (type locality: Perú: Madre de Dios: Rio Manu, Pakitza, 11°56°47'S 071°17°00'W, 356m). An updated key to the genera and subgenera of Tachyina occurring in the New World is provided, with accompanying illustrations.

## Abstract (es)

La clasificación de la subtribu Tachyina (Carabidae: Trechitae: Bembidiini) se revisa luego de diversidad que ha sido nuevamente descrita del Centro y Sur América. Aquí se describen tres géneros nuevos (*Tachyxysta* **género nuevo**, *Stigmatachys* **género nuevo**, *Nothoderis* **género nuevo**), dos subgéneros nuevos del *Meotachys* (*Scolistichus* **subgénero nuevo**, *Hylotachys* **subgénero nuevo**), y dos subgéneros nuevos del *Elaphropus* (*Ammotachys* **subgénero nuevo**, *Idiotachys* **subgénero nuevo**). Dos nombres taxonómicos previamente sinonimizados con *Polyderis* (*Polyderidius* Jeannel, 1962) y *Elaphropus* (*Nototachys* Alluaud, 1930) aquí son elevados a los niveles de género y subgénero, respectivamente. Se reconocen ochos espécies nuevos: *Tachyxysta howdenorum* (localidad tipo: México: Chiapas: El Aguacero, 680m); *Elaphropus marchantarius* (localidad tipo: Brazil, Amazonas, Rio Solimões, Ilha de Marchantaria), *Elaphropus acutifrons* (localidad tipo: Brazil: Pará, Santarém) and *Elaphropus occidentalis* (localidad tipo: Perú: Loreto, Pithecia, 74°45'W 05°28'S); *Stigmatachys uvea* (localidad tipo: Perú: Loreto: Campamento San Jacinto, 2°18.75'S, 75°51.77'W, 175–215m); and *Meotachys riparius* (localidad tipo: Colombia: Amazonas: Leticia, 700 ft), *Meotachys ballorum* (localidad tipo: Brazil: Amazonas, Rio Negro Cucui), and *Meotachys rubrum* (localidad tipo: Perú: Madre de Dios: Rio Manu, Pakitza, 11°56°47'S 071°17°00'W, 356m). Una clave actualizada con illustraciones de los géneros y subgéneros del Tachyina que ocurren en el Nuevo Mundo está incluída.

A classificação da subtribo Tachyina (Carabidae: Trechitae: Bembidiini) está revisto à luz da diversidade recém-descrita da América Central e do Sul. Aqui são descritos três novos gêneros (*Tachyxysta* **novo gênero**, *Stigmatachys* **novo gênero**, *Nothoderis* **novo gênero**), dois novos subgêneros de *Meotachys* (*Scolistichus* **novo subgênero**, *Hylotachys* **novo subgênero**), e dois novos subgêneros de *Elaphropus* (*Ammotachys* **novo subgênero**, *Idiotachys* **novo subgênero**). Dois nomes taxonômicos anteriormente sinonimizadas com *Polyderis* (*Polyderidius* Jeannel, 1962) e *Elaphropus* (*Nototachys* Alluaud, 1930) são elevada aos níveis de gênero e subgênero, respectivamente. Oito espécies são reconhecidas: *Tachyxysta howdenorum* (localidade tipo: México: Chiapas: El Aguacero, 680m); *Elaphropus marchantarius* (localidade tipo: Brazil, Amazonas, Rio Solimões, Ilha de Marchantaria), *Elaphropus acutifrons* (localidade tipo: Brazil: Pará, Santarém) and *Elaphropus occidentalis* (localidade tipo: Perú: Loreto, Pithecia, 74°45'W 05°28'S); *Stigmatachys uvea* (localidade tipo: Perú: Loreto: Campamento San Jacinto, 2°18.75'S, 75°51.77'W, 175–215m); and *Meotachys riparius* (localidade tipo: Colombia: Amazonas: Leticia, 700 ft), *Meotachys ballorum* (localidade tipo: Brazil: Amazonas, Rio Negro Cucui), and *Meotachys rubrum* (localidade tipo: Perú: Madre de Dios: Rio Manu, Pakitza, 11°56°47'S 071°17°00'W, 356m). Também está incluída uma chave atualizada com ilustrações dos gêneros e subgêneros de Tachyina que ocorrem no Novo Mundo.

## Introduction

The cosmopolitan carabid subtribe Tachyina includes about 800 described species. In the Americas, tachyine diversity is greatest in the tropics, with species documented from a wide variety of habitats (riparian, hypogean, arboreal, corticolous, myrmecophilous, etc.). Detailed accounts of New World tachyine natural history are provided in previous publications by Erwin (1974b, 1991) and Adis et al. (1986).

**Figure 1.**
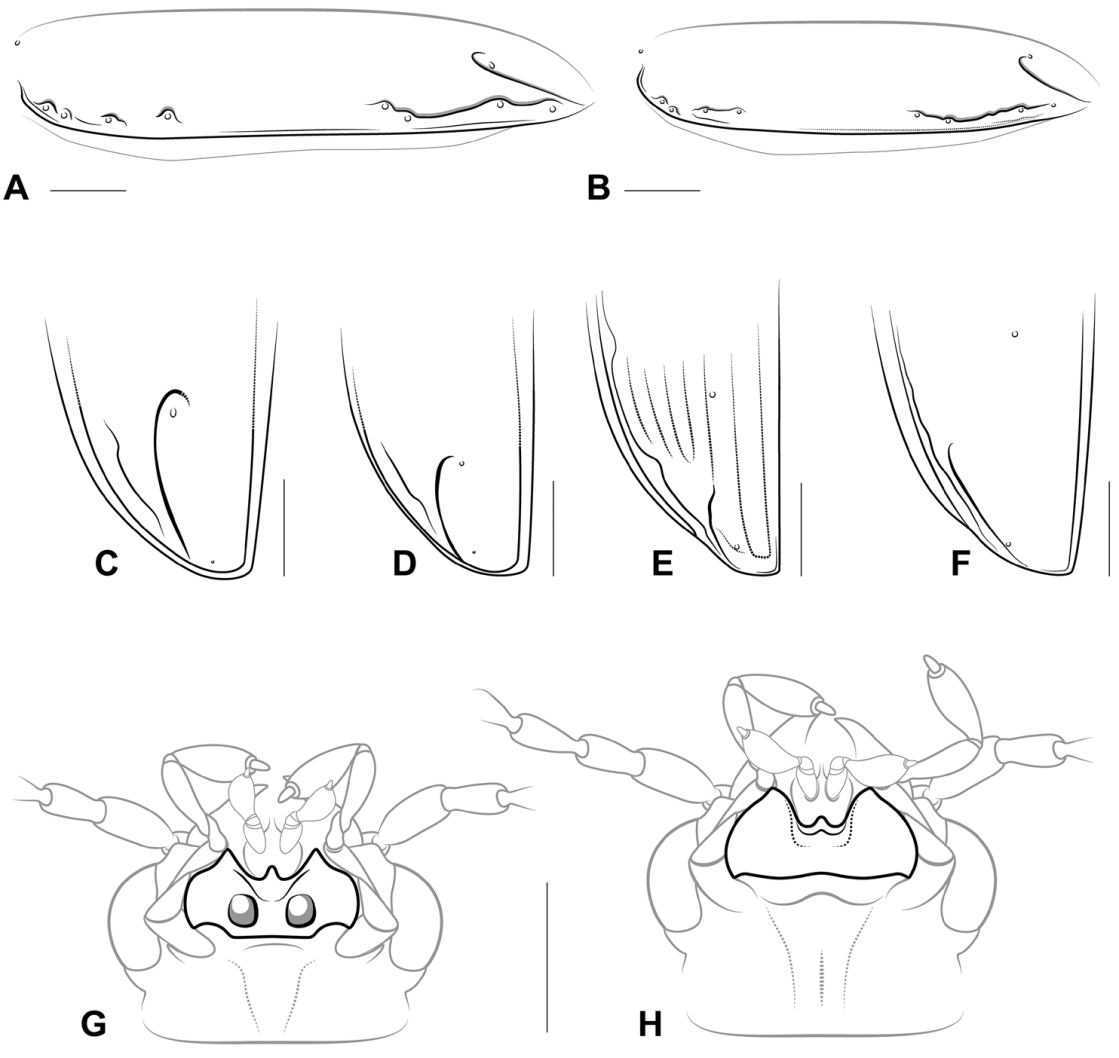
Basic characters for generic and subgeneric level identification of Tachyina. **A–B** Left elytron, lateral view, illustrating positions of elytral ombilicate setae and form of the 8^th^ elytral interneur (other interneurs omitted) **A** *Paratachys fulvicollis*; **B** *Tachys vittiger* **C–F** Elytral apex **C** *Paratachys fulvicollis* **D** *Tachys vittiger*. Scale bar 0.25 mm **E** Elaphropus (Ammotachys) marchantarius **F** *Tachyxysta howdenorum* **G–H** Head, ventral view, illustrated to show mentum **G** Meotachys (Scolistichus) riparius **H** Elaphropus (Barytachys) nebulosus. Scale bars = 0.25 mm.

**Figure 2.**
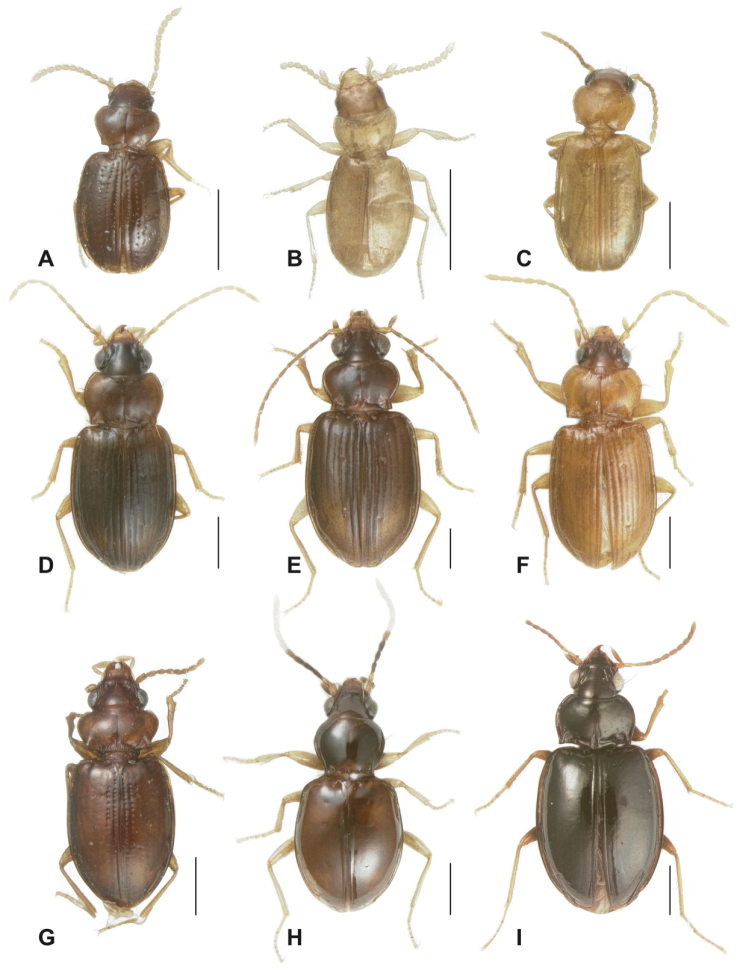
New Tachyina. **A** *Stigmatachys uvea* (holotype, ♀), Perú, Loreto, Campamento San Jacinto **B** *Polyderidius* sp., México, San Luis Potosí; **C** *Nothoderis rufotestacea*, USA, Arizona, Cochise Co. **D** Meotachys (Hylotachys) ballorum (paratype, ♀), Brazil, Amazonas, Tapurucuara **E** Elaphropus (Ammotachys) marchantarius (paratype, ♂), Venezuela, San Carlos de Rio Negro **F** Meotachys (Scolistichus) riparius (paratype, ♀), Brazil, Amazonas, Rio Solimões, Ilha de Marchantaria; **G** Elaphropus (Idiotachys) acutifrons (holotype, ♀), Brazil, Santarem **H** Elaphropus (Nototachys) occidentalis (paratype, ♂), Perú, Madre de Dios, Pakitza **I** *Tachyxysta howdenorum* (holotype, ♂), México, Chiapas, El Aguacero. Scale bars = 0.5 mm.

Among Bembidiini, tachyines are well-defined morphologically. All but a few tachyines have at least a trace of an elytral apical recurrent groove, which can vary in form (Fig. 1C–F). The apicolaterally notched protibiae (visible in Fig. 2D–G, I) of all Tachyina s. str. easily distinguish them from their closest relatives, the Xystosomina, which possess simpler, truncate protibiae (Erwin 1994). The mentum of a tachyine beetle may either bear paired foveae (Fig. 1G) or lack these structures (Fig. 1H), and though major taxonomic groups of tachyines can be classified broadly according to this character (see Figure 5 in Ortuño and Arillo 2015), it is unlikely to be phylogenetically informative. The scope of this review is limited to the Tachyina of the New World, including brief diagnoses of all New World genera, as well as descriptions of new genera and new taxa that serve to clarify the boundaries and definitions of existing genera. Many additional species await description. The genus *Meotachys* Erwin, 1974 includes a small number of undescribed species (including the largest tachyine known from the New World) in addition to those representing the two new subgenera described below. This genus is of special interest due to its diversity, its potential key phylogenetic position, and the discovery of several unique external characters. Known species of *Meotachys* vary remarkably in size and form, though all share a distinctively shaped apical portion of the 8^th^ elytral interneur. *Meotachys* is unusually heterogeneous for a group of its size; alternatively, the species richness and diversity of this group may be much larger than that which is currently represented in collections. Distributed from México to central Brazil, *Meotachys* species are associated with silty river margins, understory bamboo thickets (Erwin 1991), and the seasonal white water (várzea) and black water (igapó) inundation forests that occur throughout the Amazon basin.

Among the “non-bifoveate’ Tachyina (Ortuño and Arillo 2015), the speciose and morphologically diverse genus *Elaphropus* is defined by a lack of features diagnostic for other genera (e.g., *Micratopus*, *Lymnastis*, *Anomotachys*, *Tachyta*, etc.) rather than by a convincing set of synapomorphies. Despite the suspect monophyly of *Elaphropus*, previous authors have indicated with varying degrees of certainty that its relatively well-defined subgeneric level groups belong to a “phyletic line” (Sciaky and Vigna-Taglianti 2003). Assignment of several new species described below to *Elaphropus* is done so according to historical precedent and is somewhat tentative, as these taxa may represent separate genera pending further study. The development of a molecular phylogeny of the subtribe is in progress; with a focus on deep taxon sampling in Elaphropus (Tachyura) and allies, molecular data should help illuminate natural groups among this group’s numerous and disparate species.

## Methods

### Material examined

Codes for the institutions where type material will be deposited appear in the text as follows:

AMNH
American Museum of Natural History, New York City, N.Y., USA; Lee Herman, Curator 


CAS
California Academy of Sciences, San Francisco, CA, USA; David H. Kavanaugh, Curator 


CMNH
Carnegie Museum of Natural History, Pittsburgh, PA, USA; Robert Davidson, Collection Manager 


MZUSP
Museum of Zoology, University of Sao Paulo, Brazil; Cleide Costa, Curator 


NMNH
 National Museum of Natural History, Washington, D.C., USA; Terry L. Erwin, Curator 


OSAC
Oregon State Arthropod Collection, Oregon State University, Corvallis, OR, USA; David Maddison, Director and Curator 


UNMSM
 Museo Historia Natural, San Marcos University, Lima Perú; Gerardo Lamas, Curator 


ZSM
Bavarian State Collection of Zoology (Zoologische Staatssammlung München), Munich, Germany; Martin Baehr, Curator 


### Morphological methods: specimen preparation and imaging

DNA voucher specimens representing some of the taxa described were available from a separate project. Males were dissected following DNA extraction. Genitalia were cleared in KOH and mounted in Euparal following the procedure described by Maddison (2014). Photo references for illustrations of genitalia were obtained using a JVC KY-F75U camera-equipped Leica DM5500 B compound microscope in bright field illumination.

External structures were examined using a Leica M165 C dissecting microscope. Measurements were taken digitally using a camera-equipped Leica Z6 and the software Cartograph (Microvision). Measurements represent a range from the smallest to largest specimen examined. Abbreviations and definitions of measurements provided are listed below. Photomicrographs obtained with this system were compiled into stacked images using the photomontage software Zerene Stacker (Zerene Systems). Digital illustrations were prepared from reference photos using Adobe Creative Cloud software tools (Adobe Systems 2015).

### Descriptive terms

Morphological terms generally follow the conventions established by Erwin (1974a). Elytral ombilicate (Eo) setae are named by position according to Erwin’s (1974a) chaetotaxy system. Elytral discal (Ed) setae are simply counted and referred to in ascending order from base to apex, beginning with the scutellary seta (Ed1). Arrangement of humeral setal insertions is discussed in the key as either symmetric (with notation d(1,2) = d(3,4) indicating a distance between the first and second setae which is more or less equal to the distance between setae three and four), or asymmetric (with notation d(3,4) > d(1,2) and d(3,4) > d(2,3) indicating unequal spacing among humeral setae). Several commonly used terms are abbreviated in the key and text as follows:

i1
 elytral interneur 1 (closest to suture) 


i8
 elytral interneur 8 (closest to lateral margin) 


Ed
 elytral discal seta 


Eo
 elytral ombilicate seta 


ABL
 apparent body length (labrum to elytral apex of specimen in horizontal view) 


SBL
 standardized body length (labrum to posterior supraorbital seta + pronotum from base to apex at center line + base of scutellum to elytral apex) 


TW
 total width across widest point of both elytra 


ARG
 elytral apical recurrent groove 


Label data are listed verbatim, with label breaks denoted as follows: “label data” / “begin new line on label” // “begin second label”.

### Updated Classification and Checklist of genera and subgenera of New World Tachyina

**Genus (Subgenus) # described species occurring in the Americas**

*Moirainpa* Erwin, 1984 1

*Micratopus* Casey, 1914 5

*Lymnastis* Motschulsky, 1862 2

*Costitachys* Erwin, 1974 2

*Tachyta* Kirby, 1837

(*Tachyta*) 6

*Tachyxysta* **gen. n.** 1

*Elaphropus* Motschulsky, 1839

(*Tachyura* Motschulsky, 1862) 2

(*Barytachys* Chaudoir, 1868) 38

(*Ammotachys* **subgen. n.**) 1

(*Idiotachys* **subgen. n.**) 1

(*Nototachys* **subgen. n.**) 1

*Porotachys* Netolitsky, 1914 1

*Polyderis* Motschulsky, 1862

(*Polyderis*) 1

*Liotachys* Bates, 1871 1

*Tachysbembix* Erwin, 2004 2

*Tachys* Dejean, 1821 15

*Paratachys* Casey, 1918 50

*Polyderidius* Jeannel, 1962 8

*Stigmatachys* **gen. n.** 1

*Nothoderis* **gen. n.** 3

*Meotachys* Erwin, 1974

(*Meotachys*) 8

(*Scolistichus* **subgen. n.**) 1

(*Hylotachys* **subgen. n.**) 2

*Pericompsus* LeConte, 1851

(*Pericompsus*) 49

(*Eidocompsus* Erwin, 1974) 13

### Key to genera and subgenera of New World Tachyina

**Table:** 

1	Mentum normal for Carabidae, afoveate (Fig. 1H)	**2**
–	Mentum with pair of paramedial, rather deep circular foveae (Fig. 1G)	**13**
2(1)	Elytral intervals carinate; pronotum with longitudinal carinae	***Costitachys* Erwin, 1974**
–	Elytron and pronotum not carinate	**3**
3(2’)	Labrum bilobed, covering mandibles; elytron truncate, pubescent; ARG barely impressed or absent	**4**
–	Labrum truncate; elytron various; ARG various	**5**
4(3)	Head with single pair of supraorbital setae; male and female both with two pairs apical abdominal ventral setae, lateral pair sickle-shaped	***Micratopus* Casey, 1914**
–	Head with two pairs of supraorbital setae; apical abdominal ventrite of male with 2 longer setae, female with 4 (Old World—adventive)	***Lymnastis* Motschulsky, 1852**
5(3’)	Form depressed; ARG absent; body dorsally pubescent; eyes reduced, with a few large facets	***Moirainpa* Erwin, 1984**
–	Form convex to subdepressed; ARG visible, feebly to markedly impressed; body dorsally glabrous or (rarely) sparsely setose	**6**
6(5’)	ARG elongate, subparallel to elytral margin (Figs 5I, 1F)	**7**
–	ARG very short (Figs 4G, 4H, 5G, 5H), or elongate and arcuate toward midline and/or continuous with i3 (Figs 5F, 1E)	**9**
7(6)	Tarsal claws denticulate; prosternum plurisetose; body dorsoventrally compressed, dorsal surface coarsely microsculptured	***Tachyta* Kirby, 1837**
–	Tarsal claws simple; prosternum glabrous; body convex to subdepressed; dorsal surface various	**8**
8(7’)	Dorsal surface with coarse, isodiametric microsculpture; ARG straight; form subdepressed; color light brown; pronotum without basal excavation (Pan-tropical—adventive)	***Elaphropus yunax* (Darlington, 1939)**
–	Dorsal surface without microsculpture, except for labrum; ARG sinuate, slightly hooked anteriorly; form robust, convex (Fig. 2I); color piceous to black; basal section of pronotum with deep excavation opposite scutellum and deep, basolateral indentations (Fig. 3I)	***Tachyxysta* gen. n.**
9(6’)	Humeral series of elytral ombilicate punctures evenly spaced or symmetrically distributed, with d(1,2) = d(3,4) (Fig. 4F, 4G); elytra without macula(e); mesepisternum without fovea(e)	**10**
–	Humeral series of Eo punctures asymmetrically distributed, with Eo4 removed from closely grouped Eo1–3 such that d(3,4) > d(1,2) and d(3,4) > d(2,3) (Fig. 4H, 4I); elytra with or without macula(e); mesepisternum with or without fovea(e)	**11**
10(9)	Elytron with 8 micropunctulate interneurs; i4–7 not reaching apex and i4–5 converging apically (Figs 1E, 5F); pronotum subquadrate, margins not markedly sinuate (Fig. 3F); ARG arcuate, continuous with i3; i8 nearly complete, with basal part parallel to elytral margin, meeting or just short of reaching humeral series of setae (Fig. 4F); apical half of i8 deeply impressed, curvy, abruptly bent around Eo5+6 and Eo7 (Figs 1E, 4F, 5F)	**Elaphropus (Ammotachys) subgen. n.**
–	Elytron with 1–2 punctate interneurs (Fig. 2G), i2 effaced near apex and i3 only very faintly impressed; Pronotum wider than long, with sinuate margins (Fig. 3G); ARG very short, extended past Ed4; i8 reduced, interrupted, just visible near (but not passing through) Eo5 and 6 and between Eo7 and 8 (Figs 4G, 5G)	**Elaphropus (Idiotachys) subgen. n.**
11(9’)	Pronotum longer than wide, constricted at base (Fig. 3H); elytron with 5 discal setae, 1–3 closely grouped in basal third, 4th at apical third; apical half of antennae lighter than basal half, almost white (Fig. 2H); mesepisternum with 2 deep, circular foveae (Fig. 7A)	**Elaphropus (Nototachys) Alluaud, 1930**
–	Pronotum subquadrate to transverse and cordiform; elytron with 4 discal setae, antennae concolorous	**12**
12(11’)	i8 interrupted at middle; mesepisternum with one or more shallow fovea(e) or punctures	**Elaphropus (Barytachys) Chaudoir, 1868**
–	i8 entirely impressed, subparallel to elytral margin	**Elaphropus (Tachyura) Motschulsky, 1862**
13(1’)	Head with 3 pairs supraorbital setae (Fig. 3B); ARG (if present) often hooked anteriorly (Fig. 4B); small to minute, soft-bodied; elytra translucent, flavous, usually apically truncate and rounded (southeastern USA to Argentina) (Figs 2B, 3B, 4B, 5B)	***Polyderidius* Jeannel, 1962**
–	Head with 2 pairs supraorbital setae	**14**
14(13’)	Pronotum markedly constricted basally; i8 absent externally; form “ant-like”; apical half of antennae usually lighter than basal half, often whitish	***Liotachys* Bates, 1871**
–	Pronotum cordiform to quadrate; i8 variable in shape and completeness; overall form not as above	**15**
15(14’)	ARG elongate, very close to and parallel with elytral margin (see Fig. 1F)	***Porotachys* Netolitzky, 1914**
–	ARG varied in form (very faint to markedly impressed, short to elongate, simple or hooked), but not parallel to elytral margin (directed anteriorly toward elytral disc or closer to suture than margin) (see Figs 1C–1E, 5A–5E)	**16**
16(15’)	ARG anteriorly hooked around, into, or effaced laterad of 4th discal seta	**17**
–	ARG simple, not hooked, short to elongate	**19**
17(16)	ARG hooked into or effaced laterad of 4th discal seta (Fig. 1D), IF effaced, specimen from sea coast and with granulate microsculpture; i8 subsulcate, not incurved at Eo5–6 (Fig. 1B)	***Tachys* s. str. Stephens, 1829**
–	Hook of ARG either surrounding or produced laterad of 4th discal seta; i8 medially incurved, diverted from elytral margin at Eo5–6	**18**
18(17’)	ARG hooked around 4th discal seta (Fig. 1C); width across eyes at widest point less than greatest width of pronotum; i8 subsulcate posterior to midpoint of elytron (Fig. 1A); elytra with transverse, linear microsculpture	***Paratachys* Casey, 1918**
–	Hook of ARG produced laterad of 4th discal seta; width across eyes at widest point about equal to greatest width of pronotum; i8 shallow; surface dull, with coarse, granulate microsculpture; specimen from sea coast	***Tachysbembix* Erwin, 2004**
19(16’)	Pronotum convex, with barely rounded hind angles; i8 reduced, faintly visible apically, not redirected around elytral ombilicate setae	***Polyderis* s. str Motschulsky, 1862**
–	Pronotum shallowly convex to subdepressed, with square to acute posterior angles; i8 partially to completely impressed, apically diverted around Eo5–6, Eo7 OR interneurs deeply punctate and reduced in number	**“*Pericompsus* / *Meotachys* complex” 20**
20(19’)	Pronotum with continuous, punctate transverse impression, usually arcuate (forming crescent-shaped basal section), sometimes bilobed; i8 with conspicuous posthumeral foveae or fovea, usually at basal fourth or midpoint OR elytron with 8 entirely punctate interneurs; elytra with or without color pattern or macula(e)	***Pericompsus* 25**
–	Pronotum with punctate or subsulcate transverse impressions converging at medial furrow, forming triangular basal section (see Fig. 3A–E); i8 visible or not, without posthumeral foveae; elytron with up to 8 micropunctulate or striatiopunctate interneurs OR with 6 or fewer punctate interneurs; elytra unicolorous	**21**
21(20’)	Elytral humeri obliquely rounded (possibly brachypterous), margins serrate; elytron with at most 6 deeply punctate interneurs (Fig. 5A), i8 not visible (Fig. 4A); ARG very small, just visible at elytral apex (Figs 4A, 5A); eyes reduced; body dorsally opaque, red-brown (Peruvian Amazon) (Figs 2A, 3A, 5A)	***Stigmatachys* gen. n.**
–	Elytral humeri squarely rounded, margins smooth or serrate (Fig. 5C–E); i8 at least visible apically, medially incurved at Eo5–6 (Fig. 4C–E) and ARG rudimentary (see Fig. 1E) to distinct and markedly engraved; eyes large; degree of dorsal infuscation variable	**22**
22(21’)	Elytral interneurs distinctly punctate, fewer than 8 entirely visible	***Meotachys* s. str. Erwin, 1974**
–	Elytral interneurs micropunctulate to striatiopunctate, up to 8 entirely visible	**23**
23(22’)	Pronotum transversely cordate, margins sinuate, posterior angles prominent and slightly acute (Fig. 3E); mesepisternum with single small, deep, reniform pit (Fig. 7B); i1 deeply impressed basally at level of scutellum; ARG short, faintly impressed, not connected to i3 (Fig. 5E); interval between ARG and i8 not raised	**Meotachys (Hylotachys) subgen. n.**
–	Pronotum transversely quadrate, margins subparallel to slightly sinuate, posterior angles approximately square (Fig. 3C–D); mesepisternum without pit; i1 without deep basal impression; ARG continuous with i3 (see Fig. 1E); elytral apex with raised interval between ARG and i8	**24**
24(23’)	Elytral margin partially to entirely serrate; i8 feebly to moderately impressed from Eo5 to apex, separated from elytral margin by Eo5–8 but not markedly curved in apical half (Fig. 4C)	***Nothoderis* gen. n.**
–	Elytral margin smooth (Fig. 5D); i8 curvy and deeply impressed in apical half, abruptly bent at Eo5–6 and around Eo7 (Fig. 4D)	**Meotachys (Scolistichus) subgen. n.**
25(20)	i8 with a deep, nearly perforate fovea at or just anterior to middle of elytron; elytron with two additional subhumeral foveae of varied size; pronotum often narrowed at base; body typically elongate; elytra often with dark markings	***Pericompsus* s. str. LeConte, 1852**
–	i8 not foveate at or near middle of elytron; if foveate posterior to humerus, then fovea shallow and bearing seta OR small, perforate, and located at basal fourth near seta Eo4c; pronotum usually quadrate, with base and apex subequal in width; body typically compact, robust, unicolorous	**Pericompsus (Eidocompsus) Erwin, 1974**

**Figure 3.**
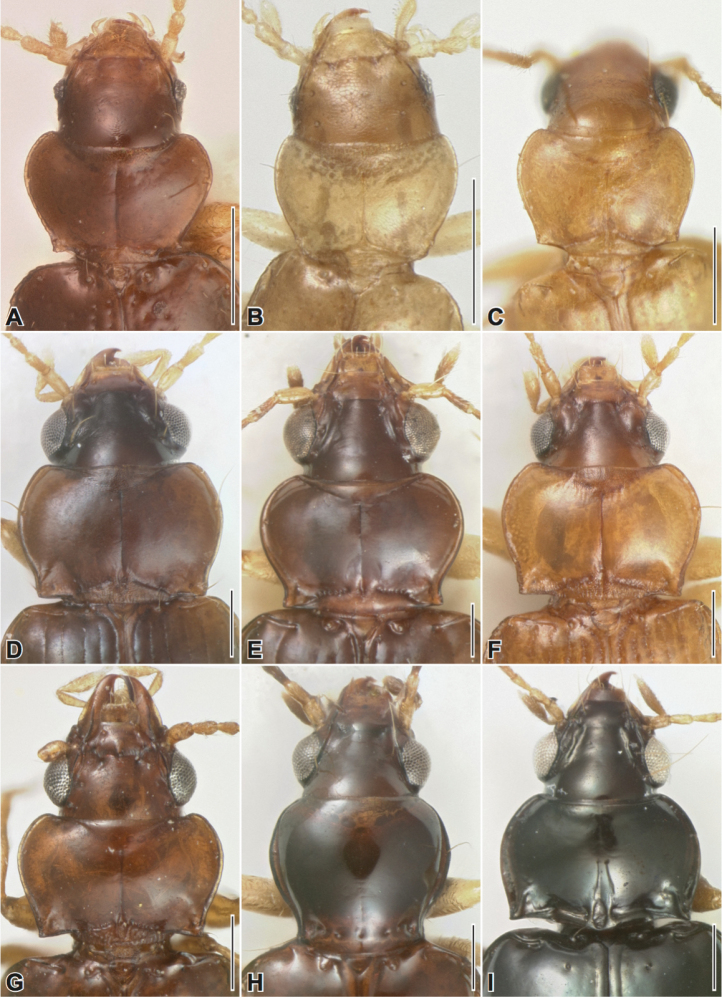
Pronotum, dorsal aspect. **A** *Stigmatachys uvea* (holotype, ♀), Perú, Loreto, Campamento San Jacinto **B** *Polyderidius* sp., México, San Luis Potosí **C** *Nothoderis rufotestacea*, USA, Arizona, Cochise Co. **D** Meotachys (Hylotachys) ballorum (paratype, ♀), Brazil, Amazonas, Tapurucuara **E** Elaphropus (Ammotachys) marchantarius (paratype, ♂), Venezuela, San Carlos de Rio Negro **F** Meotachys (Scolistichus) riparius (paratype, ♀), Brazil, Amazonas, Rio Solimões, Ilha de Marchantaria; **G** Elaphropus (Idiotachys) acutifrons (holotype, ♀), Brazil, Santarem **H** Elaphropus (Nototachys) occidentalis (paratype, ♂), Perú, Madre de Dios, Pakitza **I** *Tachyxysta howdenorum* (holotype, ♂), México, Chiapas, El Aguacero. Scale bars = 0.25 mm.

**Figure 4.**
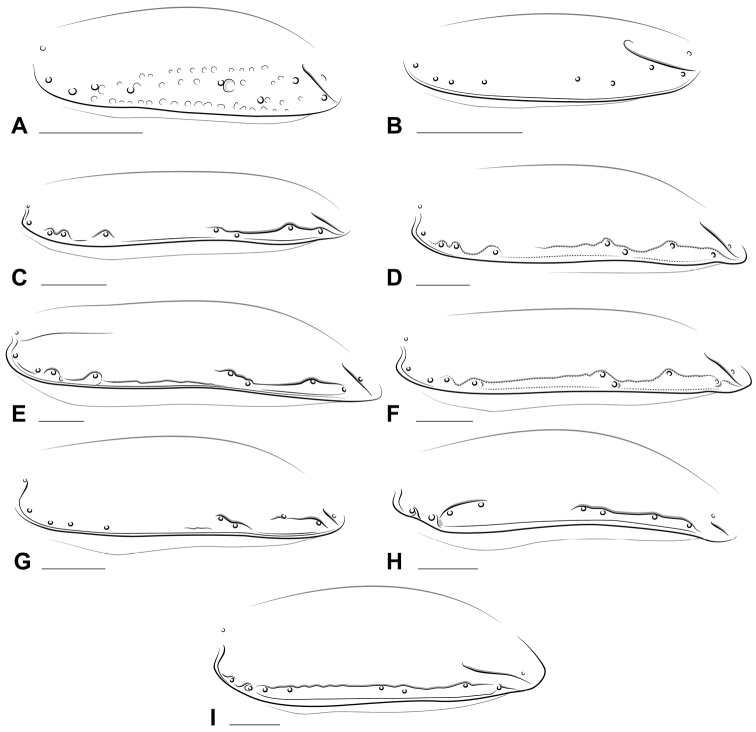
Illustrations of left elytron, lateral view, showing variation in the form of the 8^th^ elytral interneur (other interneurs not shown) among newly described taxa. **A** *Stigmatachys uvea* (holotype, ♀) **B** *Polyderidius* sp. **C** *Nothoderis* sp. **D** Meotachys (Scolistichus) riparius; **E** Meotachys (Hylotachys) ballorum; **F** Elaphropus (Ammotachys) marchantarius **G** Elaphropus (Idiotachys) acutifrons **H** Elaphropus (Nototachys) occidentalis **I** *Tachyxysta howdenorum*. Scale bars = 0.25 mm.

**Figure 5.**
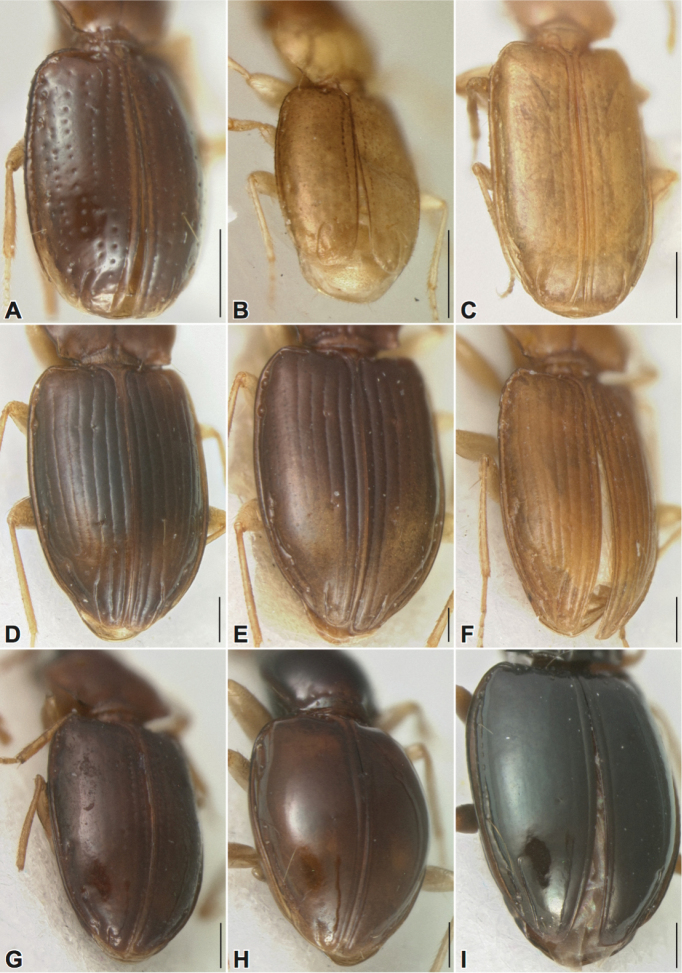
Elytral apex, left dorsal oblique view. **A** *Stigmatachys uvea* (holotype, ♀), Perú, Loreto, Campamento San Jacinto **B** *Polyderidius* sp., México: San Luis Potosí **C** *Nothoderis rufotestacea*, USA: AZ: Cochise Co. **D** Meotachys (Hylotachys) ballorum (paratype, ♀), Brazil, Amazonas, Tapurucuara **E** Elaphropus (Ammotachys) marchantarius (paratype, ♂), Venezuela, San Carlos de Rio Negro **F** Meotachys (Scolistichus) riparius (paratype, ♀), Brazil, Amazonas, Rio Solimões, Ilha de Marchantaria **G** Elaphropus (Idiotachys) acutifrons (paratype, ♀), Brazil, Santarem **H** Elaphropus (Nototachys) occidentalis (paratype, ♂), Perú, Madre de Dios, Pakitza **I** *Tachyxysta howdenorum* (holotype, ♂), México, Chiapas, El Aguacero. Scale bars = 0.25 mm.

**Figure 6.**
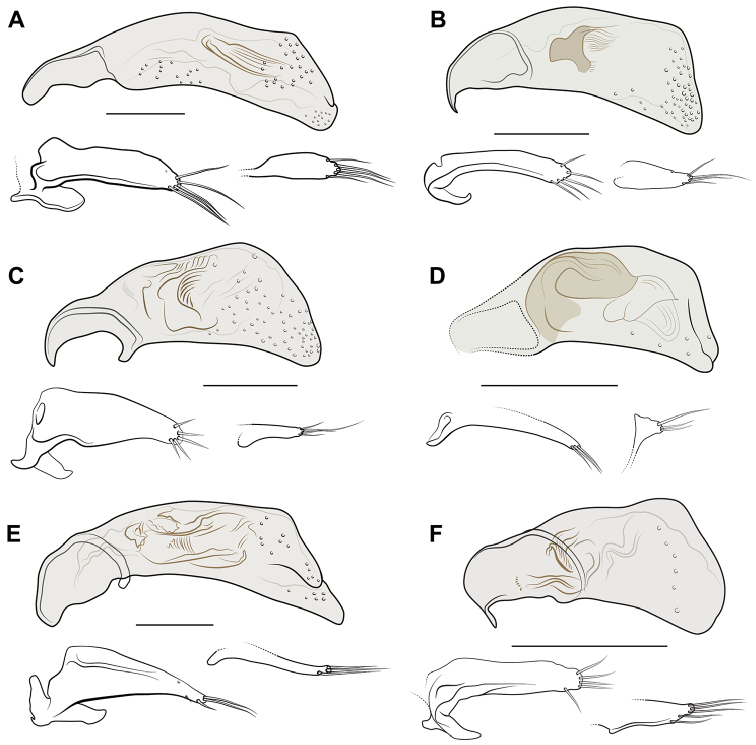
Illustrations of aedeagus, left lateral aspect, with parameres (left and right) shown below median lobe. **A** Meotachys (Hylotachys) ballorum, DRM voucher DNA2854 **B** *Nothoderis* sp., DRM voucher DNA2870 **C** *Nothoderis* sp., DRM voucher DNA2935 **D** *Polyderis laeva*, DRM voucher DNA2913 **E** *Tachyxysta howdenorum*, DRM voucher 4881 **F** *Nothoderis rufotestacea*, DRM voucher DNA0718. Scale bars = 0.1 mm.

**Figure 7.**
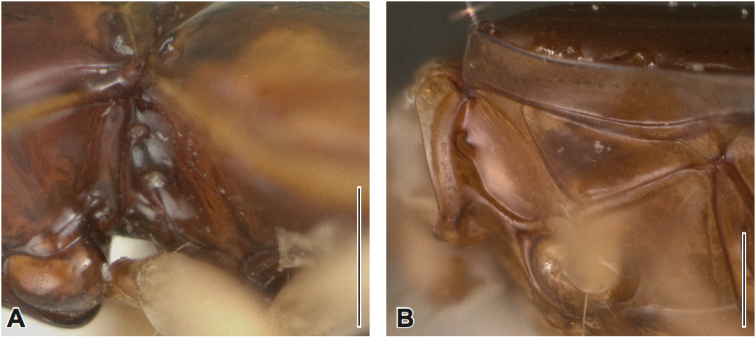
Mesepisternal pits of new Tachyina. **A** Elaphropus (Nototachys) occidentalis (paratype, ♂) Perú, Loreto **B** Meotachys (Hylotachys) ballorum (paratype, ♀), Brazil, Amazonas, Rio Demiti. Scale bars = 0.25 mm.

## Diagnoses and descriptions of genera and subgenera of New World Tachyina

### Genus Moirainpa

Taxon classificationAnimaliaColeopteraCarabidae

Erwin, 1984

#### Type species.

*Moirainpa amazona* Erwin, 1984.

#### Diagnosis.

Mentum without foveae; eyes reduced and pubescent, with a few large facets; labrum not covering mandibles; foretibia notched apicolaterally; elytron entire, with serrate humeral margin; ARG absent; body subdepressed, pubescent. ABL = 1.0 mm.

#### Distribution.

Known from várzea white water inundation forests of the upper to middle Amazon River drainage (Erwin 1984).

### Genus Micratopus

Taxon classificationAnimaliaColeopteraCarabidae

Casey, 1914

#### Type species.

*Blemus aenescens* (LeConte, 1848).

#### Diagnosis.

Mentum without foveae; labrum bilobed, covering mandibles; head with single pair of supraorbital setae; elytra truncate; terminal abdominal ventrite of both male and female with four long setae, the lateral pair sickle-shaped. ABL = 1.2–3.0 mm.

#### Distribution.

Often abundant at lights, this speciose and underdescribed genus is known from southern North America to northern South America and the Caribbean (Erwin 1991, Erwin et al. 2002).

### Genus Lymnastis

Taxon classificationAnimaliaColeopteraCarabidae

Motschulsky, 1862

#### Type species.

*Lymnaeum indicum* (Motschuslky, 1851).

#### Diagnosis.

Mentum without foveae; labrum bilobed, covering mandibles; head with two pairs of supraorbital setae; elytra slightly truncate; terminal abdominal ventrite of male with two long, straight setae, female with four long, straight setae; body densely to sparsely setose.

#### Distribution.

This Old World genus is adventive in Hawaii and the Caribbean (Erwin et al. 2002).

### Genus Costitachys

Taxon classificationAnimaliaColeopteraCarabidae

Erwin, 1974

#### Type species.

*Costitachys inusitatus* Erwin, 1974.

#### Diagnosis.

Mentum without foveae; foretibia with apicolateral notch; body subdepressed, flavotestaceous, shiny; head with single pair of supraorbital setae; basal protarsomere of male dilated, medially dentiform; head (3), pronotum (5), and elytron (8) with prominent longitudinal carinae; ABL = 1.7–2.6 mm.

#### Distribution.

Known from sandy riparian habitats throughout the Amazon basin, from the eastern Andes of Ecuador and Perú to the Atlantic coast of South America (Erwin 1974; Erwin and Kavanaugh 1999, 2007).

### Genus Tachyta

Taxon classificationAnimaliaColeopteraCarabidae

Kirby, 1837

#### Type species.

*Tachyta picipes* Kirby, 1837.

#### Diagnosis.

Elongate, subdepressed; mentum lacking foveae; dorsal surface (excluding Old World taxa) with coarse, isodiametric microsculpture; prosternum plurisetose; tarsal claws denticulate; basal two protarsomeres of male dilated, medially dentiform; ARG elongate, slightly hooked anteriorly, close and subparallel to lateral margin of elytron. ABL = 1.8–3.3 mm.

#### Distribution.

Widely distributed from the boreal Nearctic to Central America and the Caribbean, associated with fallen logs (Erwin 1975).

### Genus Tachyxysta

Taxon classificationAnimaliaColeopteraCarabidae

Boyd & Erwin gen. n.

http://zoobank.org/316DFAA8-D484-4AD1-A2B5-1380C9CED42D

#### Type species.

*Tachyxysta howdenorum* Boyd & Erwin, sp. n.

#### Diagnosis.

Pronotum with distinctly inflated basal section separated from pronotal disc by subsulcate transverse impression; basal section interrupted at midpoint by prominent, deep excavation opposite scutellum; overall form robust, convex.

#### Description.

Size. ABL = 2.4–2.5 mm; SBL = 2.5–2.7 mm; TW = 1.15–1.25 mm.

Form. Compact, robust, convex.

Color. Dorsally piceous, unicolorous (Fig. 2I); antennae and legs lighter, rufotestaceous except for darker basal half of coxae; dorsally glabrous and without microsculpture except for labrum.

Head. Two pairs of supraorbital setae within channeled longitudinal frontal furrows; frons not raised between furrows, often with subtle transverse wrinkles (Fig. 3I); mentum without foveae.

Prothorax. Base of pronotum (Fig. 3I) with deep lateral depression near posterior angle; posterior angle of pronotum raised, prominent; basal section of pronotum convex, interrupted by deep medial excavation opposite scutellum; male without dilated basal protarsomere(s); protibia notched apicolaterally (Fig. 2I); tarsal claws simple, not denticulate.

Pterothorax.Elytral margin reflexed; i1 entire, subsulcate or faintly impressed; i2–i7 not visible; i8 striatiopunctate from humerus to Eo5, apically subsulcate (Fig. 4I); recurrent groove elongate, slightly sinuate, subparallel to elytral margin, and recurved anteriorly (Fig. 5I); surface without spots; elytral ombilicate setae 2, 6, and 8 more than twice as long as next longest seta.

Genitalia. male aedeagus robust, elongate, with unequally sized, apically 3- or 4-setose parameres (Fig. 6E).

#### Distribution.

The Mexican specimens were collected near or in El Ocote Preserve; the Honduran specimen was collected in an area near Comayagua National Park. Based on collection data from a limited number of specimens, *Tachyxysta howdenorum* may be restricted to higher altitudes.

#### Derivation of name.

Feminine. Derived from *Tachys*, the nominate genus of the subtribe Tachyina, and the Greek *xustos* (=“smooth/polished”), in reference to this species’ unmicrosculptured, glabrous dorsal surface and alluding to its general resemblance to some members of the subtribe Xystosomina, particularly those of the genus *Erwiniana*.

### Tachyxysta howdenorum sp. n.

Taxon classificationAnimaliaColeopteraCarabidae

http://zoobank.org/62D8132A-524B-4C03-A219-3BC8B2FEBB3A

Figs 2I , 3I , 4I , 5I , 6E

#### Type material.

Holotype: male (UASM) with following label data: “MEXICO. Chiapas, / El Aguacero, 16 km / W Ocozocoautla / 680m 5.[?]13.VI.1990 / H. & A. Howden FIT”. Paratypes: 6 (2 male, 4 female) in CNC, FSCA, UASM, from type locality [1♀, UASM], “MEX.,Jnct.Rts. / 190&195,Chis. / VI-6-1969 / J.M.Campbell” [2♂, one with second label, “At Black / Light”, CNC], “HONDURAS / Comayagua Dept. / Rancho Chiquito / Km 64 / 29 May 1964 // Blanton, Broce, / & Woodruff Coll. / Light:UV, trap” [1♀, FSCA], “Jct.Hwys 190-195, / Chis.Mex.VI.6, / 1969 H.F.Howden” [2♀, UASM].

#### Type locality.

México: Chiapas: El Aguacero.

#### Description.

Size, form, color, head, prothorax, pterothorax, abdomen, genitalia, and distribution as in description of the genus.

#### Derivation of specific epithet.

The patronym *howdenorum* honors Henry and Anne Howden, collectors of the holotype. The Howdens collected several examples included in the type series of *Tachyxysta howdenorum* two decades apart in different locations.

#### Remarks.

Despite its superficial resemblance to the genus *Xystosomus*, *Tachyxysta howdenorum* possesses a combination of characters that support its placement among the Tachyina but discourage its membership in any previously described tachyine genus; *Tachyxysta howdenorum* has an apicolaterally notched protibia and an apical recurrent groove reminiscent of *Tachyta*, but lacks the denticulate tarsal claws diagnostic for that genus (Erwin, 1975).

### Genus Elaphropus

Taxon classificationAnimaliaColeopteraCarabidae

Motschulsky, 1839

#### Type species.

*Elaphropus caraboides* Motschulsky, 1862.

#### Diagnosis.

Mentum lacking foveae; claws simple; prosternum glabrous; elytron with 2 to 8 visible interneurs, complete or not, and variable in form; i8 impressed entirely or effaced at midpoint or visible only near apex; ARG short, straight or medially arcuate or if elongate and parallel to elytral margin, not recurved anteriorly.

### Subgenus Tachyura

Taxon classificationAnimaliaColeopteraCarabidae

Motschulsky, 1862

#### Type species.

*Elaphrus quadrisignatus* Duftschmidt, 1812.

#### Diagnosis.

i8 impressed from base to apex, subparallel to elytral margin, inserted anteriorly between Eo2 and Eo3; humeral series of elytral ombilicate setal insertions asymmetrically distributed, with Eo4 removed from closely grouped Eo1–3 such that d(3,4) > d(1,2) and d(3,4) > d(2,3); ARG short, straight to medially arcuate or elongate, straight, and parallel to elytral margin (*Elaphropus yunax*).

#### Distribution.

Old World, Australia; adventive in the Americas.

### Subgenus Barytachys

Taxon classificationAnimaliaColeopteraCarabidae

Chaudoir, 1868

#### Type species.

*Bembidium incurvum* Say, 1834.

#### Diagnosis.

i8 visible near base and apex, middle section effaced; humeral series of elytral ombilicate setal insertions asymmetrically distributed, with Eo4 removed from closely grouped Eo1–3 such that d(3,4) > d(1,2) and d(3,4) > d(2,3); ARG short, straight or medially arcuate.

#### Distribution.

North and Central America, the Caribbean islands.

### Subgenus Ammotachys subgen. n.

Taxon classificationAnimaliaColeopteraCarabidae

http://zoobank.org/2A384793-FA57-4E0B-823B-A16D69167739

#### Type species.

*Elaphropus marchantarius* sp. n.

#### Diagnosis.

Mentum lacking foveae; elytron (Fig. 5F) with 8 micropunctulate interneurs; elytral humeral margin serrate; basal half of i8 (Fig. 4F) parallel to elytral margin, meeting or nearly reaching humeral series of setal insertions; humeral setae symmetrically distributed; apical half of i8 curvy, abruptly bent around Eo5+6 and Eo7; apical recurrent groove (Fig. 1E) rudimentary, continuous with i3.

#### Description.

Size. ABL = 2.25–2.8 mm; SBL = 2.35–2.9 mm; TW = 0.95–1.15 mm

Form. Elongate, parallel-sided, subdepressed.

Color. Uniformly yellow-brown to flavous.

Microsculpture. Head and anteromedial part of pronotum with coarse, scaly, isodiametric microsculpture; remainder of pronotum and elytron with linear, transverse microsculpture.

Head. Mentum without foveae.

Prothorax. Basal section of pronotum (Fig. 3F) triangular, rugose, with basal transverse impressions not well-defined; pronotum with dark, medial furrow that does not reach anterior margin; pronotal furrow with shallow basal excavation; convergent transverse impressions barely visible along anterior margin of pronotum; basal protarsomere of male with prominent medial dentiform expansion.

Pterothorax. Elytral margin serrate; humeral margin (Fig. 4F) with four symmetrically spaced setal insertions; elytron with 8 micropunctulate interneurs; i4–7 not reaching apex and i4–5 converging apically (Fig. 1E); basal half of i8 parallel to elytral margin, meeting or nearly reaching humeral series of setae; apical half of i8 deeply impressed, abruptly curved around Eo5+6 and Eo7 and somewhat deviated medially from elytral margin; ARG (Fig. 1E) rudimentary, continuous with i3.

Genitalia. Not examined.

#### Distribution.

Widely distributed in the Amazon basin. Known from several localities along the Rio Negro (S. Venezuela), Rio Solimões (S. Colombia and Pará, Brazil), and their confluence, and the Rio Xingu (NE Mato Grosso, Brazil).

#### Derivation of name:

Masculine. Greek noun, *ammos* (= “sand”), in reference to the habitat and coloration of the known species of this genus, and *Tachys*, the nominate genus of the subtribe Tachyina.

#### Remarks.

Though these beetles are tentatively placed within *Elaphropus* due to the afoveate mentum, their remarkable (though perhaps homoplasious) resemblance to the foveae-bearing species Meotachys (Scolistichus) riparius, calls into question the long-assumed taxonomic value and phylogenetic distribution of this character. These two species have similarly broad, pan-Amazonian, apparently overlapping distributions. Molecular data should help to clarify whether their shared morphologies are due to convergence of separate lineages or the loss or gain of foveae within a lineage.

### Elaphropus (Ammotachys) marchantarius sp. n.

Taxon classificationAnimaliaColeopteraCarabidae

http://zoobank.org/DEB2BADC-BEEC-430D-B80D-735BB7407ADC

Figs 1E , 2F , 3F , 4F , 5F

#### Type material.

Holotype: male (NMNH) with following label data: “BRAZIL/AM-(Rio Solimões) / Ilha de Marchantaria / 59°58'W 3°15'S;Várzea / J. Adis leg. 22 I 1982”. Paratypes: 8 (6 male, 2 female) in NMNH, ZSM from “BRAZIL/AM-(Rio Solimões) / Ilha de Marchantaria / 59°58'W 3°15'S;Várzea / J. Adis leg. 1.10.81” [1♂, NMNH], “BRAZIL/AM-(Rio Solimões) / Ilha de Marchantaria / 59°58'W 3°15'S;Várzea / J. Adis leg 20-2.[?] [handwritten] 1989A” // Lago Camaleão: light trap / 3.60 m above ground on / *Macrolobium acaciifolium* / leg. C Martius/A Rebello” [1♂, NMNH], handwritten label: “Jacareacanga / Para Brasie / XII-1968 / M. Alvarenga” [1♂, NMNH], “VENEZUELA, T.F.Amaz. / San Carlos de Rio / Negro, 23 Jan. 1985 / P. & P. Spangler, / R.Faitoute,W.Steiner” [1♂, NMNH], “LETICIA,Amazonas / Colombia 700 ft. / Feb.23-Mar.2/74 / H. & A. Howden” [2♂, UASM], “BRAZIL/AM-(Rio Solimões) / Ilha de Marchantaria / 59°58'W 3°15'S;Várzea / J. Adis leg. 22.XII 1981[?]” [1♀, NMNH], “Jacaré P.N.Xingu / M.Grosso- Bras. / XI.1961 / leg.M.Alvarenga” [1♀, ZSM].

#### Type locality.

Brazil: Amazonas: Rio Solimões, Ilha de Marchantaria, 59°58'W 3°15'S.

#### Description.

Size, form, color, microsculpture, head, prothorax, mesothorax, and distribution as in description of the subgenus.

#### Derivation of specific epithet.

The specific epithet *marchantarius* is a toponym referring to Ilha de Marchantaria, the collection locality of the majority of type material, located near Manaus, Brazil.

### Subgenus Idiotachys subgen. n.

Taxon classificationAnimaliaColeopteraCarabidae

http://zoobank.org/F61DBD6D-C37B-4643-AA9E-15632116072C

#### Type species.

*Elaphropus acutifrons* sp. n.

#### Diagnosis.

Mentum without foveae; head with prominent keel-like frontoclypeal carina; elytral interneurs punctate, incomplete in length, and reduced in number; i8 visible only in apical half, interrupted and reduced.

#### Description.

Size. ABL = 2.15–2.2 mm; SBL = 2.25–2.325; TW = 0.95–1.0 mm.

Form. (Fig. 2G) Oval, compact, somewhat dorsoventrally compressed.

Color. Dark reddish brown, glabrous with some small punctules.

Microsculpture. Apparently absent, but difficult to see due to specimen condition.

Head. Mentum without deep foveae but with very faint, shallow impressions at base; frons (Fig. 3G) without longitudinal furrows but with keel-like medial carina between deep lateral depressions at clypeus; margin above antennal insertion prominent, with deep sinuate groove extending to midpoint of eye; labrum coarsely microsculptured; gula densely pitted.

Prothorax. Pronotum (Fig. 3G) transversely cordiform with sinuate, somewhat reflexed margins; pronotum nearly twice as wide as long at widest point; basal section sculpted with deep longitudinal wrinkles; posterior angles prominent, square, upturned; transverse impression dividing basal section from pronotal disc punctate, interrupted medially by deep, narrow excavation; medial furrow emerging from basal excavation, not reaching anterior margin; anterior transverse impressions lightly impressed, medially convergent; prosternum sulcate, with pair of prominent longitudinal ovoid bumps; procoxae separated by broad, apically rounded prosternal process; protibial apicolateral notch (Fig. 2G) oblique, rounded, not abrupt as is typical across Elaphropus, not bearing spine.

Pterothorax. Mesepisternum neither foveate nor perforate; elytral interneurs 1–2 punctate, with second effaced near apex; i3 only very faintly impressed (Figs 2G, 5G); ARG very short, extending past Ed4; i8 (Fig. 4G) interrupted, partially impressed, just visible near (but not passing through) Eo5 and 6, and between Eo7 and 8; elytron with 9 ombilicate and 4 discal setae; humeral setal insertions widely spaced along basal third of elytral margin.

Genitalia. Not examined.

#### Distribution.

Known only from the type locality of Santarém, Pará, Brazil.

#### Derivation of name:

Masculine. From the Greek adjective *ídios* (=“self/peculiar”), in reference to its unique combination of characters, and *Tachys*, the nominate genus of the subtribe Tachyina.

#### Remarks.

Based on its afoveate mentum and apicolaterally notched protibiae, the only known species of *Idiotachys* is considered to be part of the greater *Elaphropus* complex. The overall proportions, reduced and punctate elytral strial interneurs, reduced 8th interneur, and arrangement of humeral elytral ombilicate setae diagnostic for the species described below preclude its placement in any existing *Elaphropus* subgenus.

 These, along with unique external characters, support *Idiotachys* as a lineage distinct from either *Tachyura* or *Barytachys*, the two subgenera it most closely resembles.

### Elaphropus (Idiotachys) acutifrons sp. n.

Taxon classificationAnimaliaColeopteraCarabidae

http://zoobank.org/9DDB4A8E-DE04-4868-805A-3F02903391E6

Figs 2G , 3G , 4G , 5G

#### Type material.

Holotype: female (AMNH) with following label data: “Santarem”. Paratypes: 1 female in NMNH, from type locality.

#### Type locality.

Brazil: Pará, Santarém.

#### Description.

Form, size, color, head, prothorax, pterothorax, and distribution as in description of the subgenus.

#### Derivation of specific epithet.

Derived from the Latin *acutus* (=“sharp”) and *frons* (=“front”), in reference to this species’ distinctive raised frons.

### Subgenus Nototachys

Taxon classificationAnimaliaColeopteraCarabidae

Alluaud, 1930 stat. n.

#### Type species.

*Tachys comptus* Andrewes, 1922

#### Diagnosis.

Mentum without paired foveae; antennae apically lighter than at base (Fig. 2H); dorsal surface glabrous, without microsculpture; pronotum (Fig. 3H) narrowed at base, with posterior angle approximately square or projecting slightly laterad and produced slightly anterior to base of pronotum; foretibia with apicolateral notch; elytron with two pale maculae; i8 inserted basally into deep pit between Eo2 and Eo3; i8 (if complete) distant from elytral margin at midpoint, or (if interrupted) (Fig. 4H) deeply impressed basally and apically; mesepisternum (Fig. 7A) with one or two deep pits; elytron with 4 or 5 discal setae (if 5, 2 basal setae positioned close together); only i1 and i8 visibly impressed; Eo4 distant from Eo3; ARG short, simple.

#### Distribution.

Southern Africa, South Asia, and South America.

### Elaphropus (Nototachys) occidentalis sp. n.

Taxon classificationAnimaliaColeopteraCarabidae

http://zoobank.org/D1442F62-1AD0-45B4-985C-A7E913943CE9

Figs 2H , 3H , 4H , 5H , 7A

#### Type material.

Holotype: male (NMNH) with following label data: “PERU: LORETO / Pithecia 16May90 / 74°45'W 05°28'S / T.L. Erwin Coll. // At light on Launch / in evening after / sunset’. Paratypes: 12 (4 male, 8 female) in AMNH, CAS, CMNH, MZUSP, NMNH, from “PERU: LORETO 1km SW Boca / del Rio Samira Vigilante Post / No. 1 130m 31 Aug 1991 / 04°40.5'S 074°18.9'W // Treading margins of open / grassy marsh, no water / T.L. Erwin & M.G. Pogue / Lot 73” [1♂, NMNH], “Rio Cauaburi / AM, Brasil / 9.XII.1962 / J.Bechyné col.” [1♂, MZUSP], “COLOMBIA:Amazonas / Leticia, 15-VI-65 / P. R. Craig / and J. Robb” [1♂, CAS] , “PERU: MADRE DE DIOS / Pakitza, Rio Manu / 6&9Feb90 T.L. Erwin / 70°58'W 12°07'S // Treading and / quaking river edge / fine silt/sand, / sparse vegetation” [1♂, 1♀, NMNH], “PERU: MADRE DE DIOS / Pakitza, Varzea / 18&21Feb90 TL Erwin / 70°58'W 12°07'S // In leaf litter on / upper flood margin / of Cocha Chica / Tr. Pacal /57” [1♀, NMNH], “PERU: MADRE DE DIOS / Pakitza, quebrada / 06Feb90 T.L. Erwin / 70°58'W 12°07'S // Under leaves at / edge of stream / behind lab, silt” [1♀, NMNH], PERU: LORETO, San / Regis, Rio Maranon / 73°57'W 04°32'S / 04May90, dusk // river margin, at / light on Launch / T.L. Erwin Coll.” [1♀, NMNH], PERU: Madre de Dios / Rio Tambopata Res. / 30km (air) sw Pto. / Maldonato, 290m / 12°50'S 069°20'W // B.M. 1983-455 / N.E.Stork / 3.x.-15.xi.1983 / riverbank” [1♀, NMNH], “BRASIL:Barra do / Tapirape, Mato Grosso, / I:1-15:1966 / B.Malkin leg.” [1♀, CMNH], ARGENTINA,Tucuman: / 15 Km.N.Tucuman, / Rio Sali. / Dec.30,1971 833 / Lee Herman” [1♀, AMNH], handwritten: “Rep Arg / Tucuman / Ciudad 111-59” [1♀, NMNH].

#### Type locality.

Perú: Loreto, Pithecia, 74°45'W, 05°28'S.

#### Diagnosis.

Mentum without foveae; elytron smooth, with two pale maculae and 5 discal setae, Ed1–3 closely grouped in basal third (Fig. 2H); pronotum (Fig. 3H) narrowed at base; mesepisternum (Fig. 7A) with two deep pits; apical half of antennae (Fig. 2H) lighter than basal half.

#### Description.

Size. ABL = 2.0–2.4 mm; SBL = 2.4–2.7 mm; TW = 1.0–1.2 mm.

Form. (Fig. 2H) Convex, shiny, glabrous; pronotum distinctly narrowed at base.

Microsculpture. Absent except for isodiametrically microsculptured labrum.

Color. Glabrous, rufotestaceous to piceous; elytron with two pale maculae.

Head. Mentum without foveae; frons without longitudinal depressions; frontoclypeal suture with very short lateral subfoveate grooves extending posteriad; apical half of antennae abruptly lighter that basal half, nearly white in many specimens.

Prothorax. Pronotum (Fig. 3H) narrowed at base, margins sinuate; posterior angle approximately square; lateral margin of pronotum narrowly explanate, with flange about 2× wider at midpoint than at base or apex and bordered by lateral groove; lateral groove extending to posterior angle; shallow, transverse basal impressions reduced to a series of several small, shallow punctules; basal two protarsomeres of male dilated, medially dentiform.

Pterothorax. Mesepisternum (Fig. 7A) with two deep, circular pits; elytral interneur 8 (Fig. 4H) very deeply impressed from Eo2–4, completely effaced between Eo4 and Eo5, deeply impressed from Eo5 to apex; dorsal surface glabrous and without microsculpture; elytral margin smooth; elytra apically narrowed after Eo5+6; each elytron with two pale spots; i1 subsulcate, 2–7 only visible as subcuticular dots; elytron with 5 discal setae, Ed1–4 in basal ⅔, Ed5 near tip of ARG (Fig. 2H); humeral setal insertions (Fig. 4H) asymmetrically distributed, with 4th removed from 1–3; elytral ombilicate setae 2, 6, and 8 very long, 2–3× the length of the next longest ombilicate seta; ARG (Fig. 5H) short, shallow, extending just past Ed5.

Genitalia. Not examined.

#### Distribution.

Known from Perú, Brazil, and Argentina. Widespread and apparently abundant in—though not restricted to—riparian habitats.

#### Derivation of specific epithet.

Derived from the Latin *occidens* (=“west”), in reference to the New World precinctiveness of this species. This subgenus was previously only described from the Old World.

### Genus Porotachys

Taxon classificationAnimaliaColeopteraCarabidae

Netolitzky, 1914

#### Type species.

*Trechus bisulcatus* Nicolai, 1822

#### Diagnosis.

Large in size (> 4 mm); mentum bearing paired foveae; pronotum quadrate, with square to acute hind angles; base of elytra wider than pronotum; elytra round, convex, with width across elytra conspicuously greatest at midpoint; ARG elongate, straight, very close to and subparallel with lateral margin of elytron.

#### Distribution.

Palearctic—adventive in North America.

### Genus Polyderis

Taxon classificationAnimaliaColeopteraCarabidae

Motschulsky, 1862

#### Type species.

*Tachys brevicornis* Chaudoir, 1846

#### Diagnosis.

Very small (< 2 mm), mentum bearing paired foveae; pronotum convex, posterior angles squarely rounded or slightly obtuse; antennae short, submoniliform; i8 reduced, barely impressed, visible apically; ARG very short.

#### Distribution.

Old World and Australia, with a single species (*Polyderis laeva*) in the Americas.

### Genus Liotachys

Taxon classificationAnimaliaColeopteraCarabidae

Bates, 1871

#### Type species.

*Liotachys antennatus* Bates, 1871.

#### Diagnosis.

Overall form “ant-like”; antennae lighter apically than at base; pronotum pedunculate, slender at base and lacking produced hind angles; mentum bearing paired foveae; elytron smooth, with only i1 visible (subsulcate); i8 not visible; ARG short and arcuate.

#### Distribution.

Amazon basin.

### Genus Tachysbembix

Taxon classificationAnimaliaColeopteraCarabidae

Erwin, 2004

#### Type species.

*Tachysbembix sirena* Erwin, 2004.

#### Diagnosis.

Mentum bearing paired foveae; anterior tibia with apicolateral notch; dorsal surface dull, with coarse, granulate microsculpture; head and eyes large; head and pronotum subequal in greatest width; pronotum round, subcordiform; pronotal hind angles tiny, laterally produced; i8 slightly bent around Eo5–6; ARG elongate, anterior hook laterally remote from 4^th^ discal seta. ABL = 3.3–3.9 mm.

#### Distribution.

Known from shoreline habitats along the Pacific coast of Costa Rica (Erwin 2004) and undescribed species from México to Colombia (Erwin in prep).

### Genus Tachys

Taxon classificationAnimaliaColeopteraCarabidae

Dejean, 1821

#### Type species.

*Tachys scutellaris* Stephens, 1828.

#### Diagnosis.

Mentum bearing paired foveae; protibia with apicolateral notch; dorsal surface typically with isodiametric microsculpture; pronotum subquadrate; i8 not markedly diverted around Eo5–6 (Fig. 1B); ARG elongate, hooked anteriorly into or effaced laterad of Ed4 (Fig. 1D).

#### Distribution.

Typically halophilic (Erwin et al. 2002), cosmopolitan.

### Genus Paratachys

Taxon classificationAnimaliaColeopteraCarabidae

Casey, 1918

#### Type species.

*Tachys austinicus* Casey, 1918.

#### Diagnosis.

Mentum bearing paired foveae; protibia with apicolateral notch; dorsal surface typically with transverse linear microsculpture and slight iridescence; pronotum subquadrate; i8 conspicuously bent around Eo5–6 (Fig. 1A); ARG elongate, anteriorly hooked around Ed4 (Fig. 1C) (Erwin et al. 2002).

#### Distribution.

Diverse habitats, cosmopolitan.

### Genus Polyderidius

Taxon classificationAnimaliaColeopteraCarabidae

Jeannel, 1962

Figs 2B , 3B , 4B , 5B

#### Type species.

*Polyderidius rapoporti* Jeannel, 1962 by original designation.

#### Diagnosis.

Mentum with paired, circular foveae; head with three pairs of supraorbital setae (Fig. 3B), antennae submoniliform; elytral apices rounded/truncate (Fig. 2B), apical recurrent groove hooked anteriorly (Fig. 4B). Many species have reduced eyes and some are brachypterous.

#### Description.

Size. ABL = 1.0–1.2 mm; SBL = 1.1–1.3 mm; TW = 0.45–0.5 mm.

Form. Minute, delicate, dorsolaterally compressed.

Color. (Fig. 2B) Uniformly flavous, flavous with darker head, or uniformly testaceous.

Microsculpture. Varied, from coarse/scaly/isodiametric to fine/linear/transverse.

Head. Head with three pairs of supraorbital setae (Fig. 3B); mentum with paired circular or oval-shaped foveae, or with pair of shallow impressions; eyes reduced in most members; antennae submoniliform and densely setose; subapical labial palpomere conspicuously large and bulbous.

Prothorax. (Fig. 3B) Basal section of pronotum triangular; posterior angles of pronotum bluntly square to rounded and slightly obtuse; procoxae narrowly separated by apically pointed prosternal process; male without dilated basal protarsomere(s).

Pterothorax. Elytral interneurs (if visible) punctate to striatiopunctate and very faintly impressed; i1 often entire, striatiopunctate; no trace of i8; apical recurrent groove (Fig. 4B) thin, well-impressed, and hooked anteriorly; elytral apex (Fig. 2B) rounded and truncate; flight wing with fringed margin, or reduced to a minute pad.

Abdomen. Terminal ventrite with two (male) or four (female) elongated setae.

Genitalia. See Jeannel 1962.

#### Distribution.

Known from across South and Central America, México, the southeast United States (Alabama, Mississippi), Hawaii, and islands of the Caribbean (Puerto Rico, Cuba) with the greatest species diversity in the Amazon basin (Adis et al. 1986).

### Genus Stigmatachys gen. n.

Taxon classificationAnimaliaColeopteraCarabidae

http://zoobank.org/E49F39AB-8125-48DF-9125-7DA310CB72FA

#### Type species.

*Stigmatachys uvea* sp. n.

#### Diagnosis.

Mentum bifoveate; antennae submoniliform; eyes reduced; labial subulate palpomere absent or reduced; lateral margin of pronotum sinuate; elytral humerus rounded; elytral interneurs punctate and reduced in number, with punctures becoming irregular near apex; abdominal sclerites densely and irregularly punctate.

#### Description.

Size. ABL = 1.2 mm; SBL = 1.35 mm; TW = 0.6 mm.

Form. (Fig. 2A) Minute, compact, convex.

Color. Reddish brown, with lighter appendages.

Microsculpture. Mostly absent, with local patches of isodiametric microsculpture at base of head and around eyes.

Head. (Fig. 3A) Antennae submoniliform; mentum with shallow foveae; head with two pairs of supraorbital setae; eyes reduced, each with about 12 large facets; frontoclypeal suture with small lateral subfoveate depressions; frons without longitudinal depressions; margin above antennal insertion prominent, with longitudinal bead; small dark puncture between gular sutures; labial palps very small, with subulate palpomere reduced or absent.

Prothorax. (Fig. 3A) Pronotum markedly cordiform; posterior lateral margin slightly crenulate; basal section triangular and shallowly pitted/sculptured; basal transverse impressions and medial furrow sulcate, meeting at a point to form triangular basal section; prosternal process very narrow.

Pterothorax. Mesepisternum without pits or foveae; humeral angles obliquely rounded; elytra oval, convex, each elytron (Fig. 5A) with 9 ombilicate and 4 discal setae and 6 visible punctate interneurs, with punctures scattered near apex; lateral channel lined with evenly spaced punctures; i8 not visible (Fig. 4A); elytral margin serrate; elytral disc with a few short, fine setae between i4 and margin; ARG (Figs 4A, 5A) short and deeply engraved, slightly curved medially near apex, directed toward i3.

Abdomen. Ventrites densely punctate and moderately setose.

Genitalia. Not examined.

#### Distribution.

Known only from the type locality in Loreto, Perú.

#### Derivation of name:

Masculine. Derived from the Greek noun *stigma* (=“mark” or “puncture”), in reference to the coarsely punctate elytra of the lone representative of this genus, and *Tachys*, the nominate genus of the subtribe Tachyina.

### Stigmatachys uvea sp. n.

Taxon classificationAnimaliaColeopteraCarabidae

http://zoobank.org/A8FAF4A3-EEA5-4616-BCC8-DE2DD586B564

Figs 2A , 3A , 4A , 5A

#### Type material.

Holotype: female (NMNH) with following label data: “PERU: Dept. Loreto / Campamento San Jacinto / 2°18.75'S, 75°51.77'W / 6 July 1993, 175-215 m / Richard Leschen #39 / ex. rainforest berlese”. Paratypes: none.

#### Type locality.

Perú: Loreto: Campamento San Jacinto, 2°18.75'S, 75°51.77'W, 175–215m.

#### Description.

Size, form, color, microsculpture, head, prothorax, pterothorax, abdomen, and distribution as in description of the genus.

#### Derivation of specific epithet.

Derived from the Latin noun *uvea* (=“grape”), referring to the ovate shape of the elytra of the holotype in dorsal view.

### Genus Nothoderis gen. n.

Taxon classificationAnimaliaColeopteraCarabidae

http://zoobank.org/AAD450AD-FC22-45D8-93D4-B1B6F7C0090D

Figs 2C , 3C , 4C , 5C , 6B, C, F

#### Type species.

*Tachys rufotestacea* Hayward, 1900.

#### Diagnosis.

Mentum with paired circular foveae; posterior angles of pronotum square to slightly acute (Fig. 3C); elytral interneurs striatiopunctate to micropunctulate, number visible varied; elytral margin partially to entirely serrate; i8 effaced anteriad of Eo5, separated from elytral margin by Eo5–8 (Fig. 4C); apical recurrent groove slightly arcuate, moderately impressed (Fig. 5C); elytral apex with raised plicate interval between i8 and ARG.

#### Description.

Size. ABL = 1.5–2.4 mm; SBL = 1.6–2.5 mm; TW = 0.6–1.0 mm.

Form. Small to minute, compact to elongate and subdepressed.

Color. Flavotestaceous to rufotestaceous.

Microsculpture. Varied; head and pronotum usually with isodiametric microsculpture; elytra with linear/transverse microsculpture or (rarely) glabrous.

Head. Mentum bifoveate; head with two pairs of supraorbital setae.

Prothorax. (Fig. 3C) Pronotum with prominent, square to slightly acute posterior angles; basal section of pronotum triangular, with straight or curved transverse impressions meeting at base of median furrow; basal protarsomere of male dilated, medially dentiform.

Pterothorax. (Fig. 5C) Elytron with one or more striatiopunctate to micropunctulate interneurs; Eo1 at sharpest point of humeral angle; i8 (Fig. 4C) effaced anteriad of Eo5, medially curved and moderately impressed near apex, separated from margin by Eo5–8; elytral margin partially to entirely serrate, serrations inconspicuous to prominent and individually setigerous; apical recurrent groove slightly arcuate, moderately impressed, continuous with or directed toward i3; elytral apex with raised interval between i8 and ARG.

Genitalia. (Based on male genitalia dissected and examined from single individuals of four different species) Male (Fig. 6B, C, F): overall form varied, median lobe with comb-like internal sclerites; both right and left parameres wedge-shaped, stout at base; left paramere large and broad with dark, sclerotized basal hook and 5 apical setae; right paramere smaller, with 4 or 5 apical setae. Female genitalia not examined.

#### Distribution.

Known from North, Central, and Amazonian South America.

#### Derivation of name:

Feminine. Greek adjective *nothos* (=“false/spurious”), in reference to this diverse and New World-restricted group’s misleading taxonomic history, and *deris* (=“fight” (Bousquet 2012)), from the name *Polyderis*. Members of this genus were previously classified within *Polyderis* based on a lack of useful and distinctive characters owing to their diminutive size; species of *Nothoderis* are restricted to the New World and are morphologically distinct from *Polyderis* (an old world genus with one species, *Polyderis laeva*, widely distributed in North America).

#### Remarks.

Species of *Nothoderis* are united by the shape of the pronotum, course of the eighth elytral interneur, features of male genitalia, form of the elytral apical recurrent groove, and preliminary molecular evidence. Male genitalia of *Polyderis laeva* were also examined and illustrated, and differ notably from all examples of *Nothoderis* in the form of the internal sclerite(s) and parameres (Fig. 6D). *Polyderis laeva* remains the sole new world representative of *Polyderis* s.str. based on external morphology; in addition, preliminary molecular evidence suggests that *Polyderis laeva* belongs to a lineage phylogenetically distinct from that of *Nothoderis rufotestacea* and other representatives of *Nothoderis*. Rather, members of this gen. n. are affiliated with the *Meotachys*/*Pericompsus* complex (Maddison et al. in prep.).

### Genus Meotachys

Taxon classificationAnimaliaColeopteraCarabidae

Erwin, 1974

#### Type species.

*Tachys ampicollis* Bates, 1882.

#### Diagnosis.

Subdepressed to dorsally convex; testaceous to rufous; mentum bearing paired circular foveae; foretibia notched apicolaterally; basal protarsomere of male dilated, medially dentiform; mesepisternum with or without small fovea; elytral strial interneurs varied in form, punctate to striatiopunctate; i8 apically curvy, diverted medially at Eo5–6 and Eo7; ARG short and arcuate or rudimentary and continuous with i3. ABL = 1.2–4.6 mm.

#### Distribution.

Meotachys species occupy diverse habitats and are described from México to southern Brazil (Erwin 1974, 1991).

### Subgenus Scolistichus subgen. n.

Taxon classificationAnimaliaColeopteraCarabidae

http://zoobank.org/98C5C6F4-3858-46D4-81A0-24B8FC42256A

#### Type species.

*Meotachys riparius* sp. n.

#### Diagnosis.

Pronotum and elytra with fine, linear, transverse microsculpture; pronotum quadrate; mesepisternum without fovea(e); elytron with smooth margin and 8 micropunctulate interneurs, 4–7 not extended to apex; apical recurrent groove rudimentary, continuous with i3.

#### Description.

Size. ABL = 2.3–2.35; SBL = 2.475–2.55; TW = 1.05–1.075 mm.

Form. Elongate, subdepressed.

Color. (Fig. 2D) Dark rufous to piceous or bicolored, with head and pronotum lighter.

Microsculpture. Microsculpture of anterior part of head coarse, scaly, isodiametric; pronotal and elytral microsculpture transverse, linear; elytral surface with slight iridescence.

Head. Mentum bifoveate; frontoclypeal suture very faint, with shallow lateral impressions.

Prothorax. (Fig. 3D) Pronotum quadrate; basal section triangular, posterior margin with short rugae; basal transverse impressions well-defined, punctate, interrupted by shallow basal excavation; thin medial furrow emerging from basal excavation not reaching anterior margin of pronotum; anterior convergent impressions short, thin, effaced medially; basal protarsomere of male with prominent dentiform medial expansion.

Pterothorax. Elytral margins smooth; elytron (Fig. 5D) with 8 micropunctulate interneurs; i4–7 not reaching apex; i8 (Fig. 4D) curvy and deeply impressed in apical half, abruptly bent around Eo5+6 and Eo7; ARG (see Fig. 1E) rudimentary, continuous with i3; elytral apex with raised interval between i8 and ARG.

Genitalia. Not examined.

#### Distribution.

Known from localities across the Amazon basin, from the upper Rio Negro system to the Rio Napo in Ecuador and northeastern Perú, and the lower Solimões River near Manaus.

#### Derivation of name:

Masculine. From the Greek *skolios* (=“crooked”), and *stíchos* (=“line”/“row”), in reference to the diagnostic curved 8^th^ interneur.

### Meotachys (Scolistichus) riparius sp. n.

Taxon classificationAnimaliaColeopteraCarabidae

http://zoobank.org/9C5C03C9-A541-46DB-8C06-DC1CC12F4AAF

Figs 2D , 3D , 4D , 5D

#### Type material.

Holotype: male (UASM) with following label data: “LETICIA, Amazonas / Colombia 700 ft. / Feb.23-Mar.2/74 / H. & A. Howden”. Paratypes: 5 (1 male, 4 female) in NMNH, UASM, from same locality as holotype [1♀, UASM], “BRASIL Amazonas / Rio Demiti, ca. / 0°37'N 66°48'W / below “Highland / Camp” varzea for. / Sept. 11, 1978 // BRASIL EXP. / 1978 / G.E.& K.E. Ball / Collectors” [1♂, UASM], “BRASIL Amazonas / Rio Demiti, near / Little Homestead / 0°35'N 66°41'W / varzea for. Loc. 1 / Sept. 4, 1978 // BRASIL EXP. / 1978 / G.E.& K.E. Ball / Collectors” [1♀, UASM], BRASIL/AM-(Rio Solimões) / Ilha de Marchantaria / 59°58'W 3°15'S;Várzea / J. Adis leg 13-17.5 [handwritten] 1991 // Lago Camaleão: light trap / 3.60 m above ground on / *Macrolobium acaciifolium* / leg. C Martius/A Rebello” [1♀, NMNH], “ECUADOR Napo Prov. / Laguna Jatuncocha / 20 km s Nuevo Roca- / fuerte on Rio Yasuni / sweep, 8.II.1986 / A.T. Finnamore” [1♀, UASM].

#### Type locality.

Colombia: Amazonas: Leticia, 700 ft.

#### Description.

Form, color, head, prothorax, mesothorax, and abdomen as in description of the subgenus.

#### Derivation of specific epithet.

Derived from Latin *ripa* (=“river bank/edge”), in reference to the riparian habitats throughout the Amazon basin from which this species is known.

### Subgenus Hylotachys subgen. n.

Taxon classificationAnimaliaColeopteraCarabidae

http://zoobank.org/F38109B2-B8B9-436A-A715-E66EFF4EA72B

#### Type species.

*Meotachys ballorum* sp. n.

#### Diagnosis.

Mentum bifoveate, antennae long and slender, pronotal margins sinuate, mesepisternum with single small, deep, reniform pit (Fig. 7B), i1 deeply impressed at base, apical recurrent groove (Fig. 5E) short, faintly impressed.

#### Description.

Size. ABL = 2.7–3.3 mm; SBL = 2.8–3.4 mm; TW = 1.2–1.5 mm.

Form. (Fig. 2E) Elongate, elytra subdepressed to convex.

Color. Matte and dark brown to piceous or glabrous and dark red-brown.

Microsculpture. Varied.

Head. Mentum bifoveate; antennae long, about 2/3 ABL; frons (Fig. 3E) with shallow lateral depressions at clypeus extending posteriad; margin above antennal insertion with shallow groove/channel.

Prothorax. Pronotum (Fig. 3E) transversely quadrate or narrowed near base, with base and apex about equal in width, but greatest width about 1.5× as wide as narrowest width; lateral margin of pronotum markedly sinuate; anterior convergent impressions boldly impressed, not reaching medial furrow; basal transverse impressions deeply punctate, interrupted by small but deep medial excavation; basal section of pronotum opposite scutellum smooth, inflated; thin, medial furrow emerging from basal excavation not meeting anterior margin of pronotum; prosternum smooth, not sulcate.

Pterothorax. Mesepisternum with single, small, reniform pit/fovea, with opening directed slightly posteriad (Fig. 7B); elytron (Fig. 5E) with 3–6 striate interneurs; i1 complete (reaching apex), subsulcate, originating near apex of scutellum, deeply impressed at base; i3–7 incomplete or absent, i3 abruptly bent around Ed2 and Ed3; i5 (either visible as strial interneur or shallow depression) originating at tip of elytral basal margin and clearly separating elytral disc from humeral region; i8 (Fig. 4E) basally effaced and separated from margin by Eo5–8; apical recurrent groove simple, short, faintly impressed.

#### Distribution.

Known from the type locality in Ecuador, as well as 4 localities along the Rio Negro, northern Amazonas, Brazil, and southern Perú.

#### Derivation of name:

Masculine. From the Greek *hyle* (=“wood/forest”, “matter/substance”), in reference to the association of species of this genus with Amazonian inundation forest habitats and the unique suite of characters uniting the two species, and *Tachys*, the nominate genus of the subtribe Tachyina.

### Meotachys (Hylotachys) ballorum sp. n.

Taxon classificationAnimaliaColeopteraCarabidae

http://zoobank.org/5EFB9951-AD8A-49A1-90AB-8ED61241E65F

Figs 2E , 3E , 4E , 5E , 6A , 7B

#### Type material.

Holotype: male (UASM) with following label data: “BRASIL Amazonas / Rio Negro Cucui / varzea forest / Sept. 17, 1978 // BRASIL EXP. / 1978 / G.E.& K.E. Ball / Collectors”. Paratypes: 5 (2 male, 3 female) in UASM, ZSM from the type locality [1♂, UASM], “Brasilien / Tapurucuara am / Rio Negro/Amazonas / 7 11.1963 / C. Lindemann” [1♂, 1♀, ZSM], “BRASIL Amazonas / Rio Demiti, ca. / 0°53'N 66°57'W / “La Laguna” / Varzea forest / Sept. 13, 1978 // BRASIL EXP. / 1978 / G.E.& K.E. Ball / Collectors” [1♀, UASM], BRASIL Amazonas / ca. 10 km. n.e. / São Gabriel da / Cachoeira, stream / margin, forest / Sept. 20, 1978 // BRASIL EXP. / 1978 / G.E.& K.E. Ball / Collectors” [1♀, UASM].

#### Type locality.

Brazil: Amazonas, Rio Negro Cucui.

#### Description.

Head and abdomen as in description of the genus.

Size. ABL = 3.2–3.3 mm; SBL = 3.3–3.4 mm; TW = 1.4–1.5 mm.

Form. (Fig. 2E) Large, elongate, subdepressed.

Color. Uniformly dark brown and slightly iridescent.

Microsculpture. Pronotum and elytron with very fine, linear, transverse microsculpture; head with coarse, isodiametric microsculpture.

Prothorax. Pronotum (Fig. 3E) transversely quadrate, wider than long.

Pterothorax. Elytra broad and parallel-sided, narrowed beginning at apical third, 5–6 visible strial interneurs, first two complete (Fig. 2E); i5 originating at tip of elytral basal margin and separating basal elytral disc from humeral region; otherwise as in description of the genus.

Genitalia. Male (Fig. 6A): median lobe elongate, with slender brush-shaped internal sclerite; left and right parameres both broad and paddle-shaped apically, each with 5 long apical setae; right paramere smaller. Female: not examined.

#### Distribution.

Known from Brazil, Ecuador, and Colombia.

#### Derivation of specific epithet.

Patronym in honor of George and Kay Ball, who collected the major part of the type series on their 1978 expedition to Brazil.

#### Remarks.

The habitat of this species was mistakenly regarded as várzea. In fact the specimens were collected in igapó forest (Ball, personal communication, 2016)

### Meotachys (Hylotachys) rubrum sp. n.

Taxon classificationAnimaliaColeopteraCarabidae

http://zoobank.org/C153738B-61A6-4D63-B269-FBAA9498C2E7

#### Type material.

Holotype: male (NMNH) with following label data: “PERU: MADRE DE DIOS / Rio Manu, BIOLAT Biol. Sta. / Pakitza, 356m, 16 Oct. 1989 / 11°56°47'S 071°17°00'W / T.L. Erwin Trocha Pacal /21”. Paratypes: 1 female, in NMNH, from “PERU: MADRE DE DIOS / Rio Manu, BIOLAT Biol. Sta. / Pakitza, 356m, 25 June 1993 / 11°56°47'S 071°17°00'W / T.L. Erwin & F. Pfuno // Treading red-colored leaf litter / at edge of lake shore in sunny / area Tr. Gallareta Lot 524’.

#### Type locality.

Perú: Madre de Dios: Rio Manu, BIOLAT Biol. Sta., Pakitza, 11°56°47'S 071°17°00'W, 356m.

#### Description.

Head and abdomen as in description of the genus.

Size. ABL = 2.7–2.85 mm; SBL = 2.8–2.9 mm; TW = 1.2–1.3 mm.

Form. Head and prothorax slender, elytra rounded and convex.

Color. Uniformly rufotestaceous, shiny.

Microsculpture. Head, pronotum and elytron smooth, glabrous.

Prothorax. Pronotum subequal in length and width.

Pterothorax. Elytra somewhat round and convex, each with 2–3 visible strial interneurs, only i1 completely impressed; i5 position without visible stria, but with gently sloping “shelf” which originates at tip of elytral basal margin and separates basal elytral disc from humeral region.

Genitalia. Not examined.

#### Distribution.

Known only from the type locality in the Madre de Dios region of southeastern Perú.

#### Derivation of specific epithet.

Latin *rubrum* (=“red/crimson”), in reference to the deep red-brown color of this species.

Note: The holotype will be deposited in UNMSM and is currently held in trust until the completion of studies at NMNH.

### Genus Pericompsus

Taxon classificationAnimaliaColeopteraCarabidae

LeConte, 1851

#### Type species.

*Bembidium ephippiatum* Say, 1834.

#### Diagnosis.

Mentum with paired foveae; pronotum with continuous, punctate transverse impression; basal transverse impression arcuate or lobed, forming crescent-shaped or bilobed basal section; elytron either with one to two conspicuous subhumeral fovea(e) along i8 in basal fourth or with 8 entirely punctate interneurs; elytra with or without color pattern. ABL = 1.72–3.72 mm. (Erwin 1974a; Erwin et al. 2002).

#### Distribution.

Australia and the Americas.

### Subgenus Pericompsus sensu stricto

Taxon classificationAnimaliaColeopteraCarabidae

#### Type species.

*Bembidium ephippiatum* Say, 1834.

#### Diagnosis.

Typically elongate, subcylindrical in form; pronotum quadrate to narrowed in basal fourth; elytral i8 with a deep fovea at or just anterior to midpoint of elytron and two smaller, subhumeral foveae of variable size; elytral interneurs punctate to striatiopunctate; elytra usually testaceous with darker markings resembling ink blotches. ABL = 1.88–3.72 mm. (Erwin 1974a; Erwin et al. 2002).

#### Distribution.

Species of this subgenus are numerous and described from across the New World between the temperate mid-latitudes of North and South America and some of the Caribbean islands.

### Subgenus Eidocompsus

Taxon classificationAnimaliaColeopteraCarabidae

Erwin, 1974

#### Type species.

*Trechus brasiliensis* Sahlberg, 1844.

#### Diagnosis.

Broad and robust, depressed to subcylindrical in form; pronotum quadrate, subequal in width at base and apex; elytra usually unicolorous; elytral interneurs punctate; i8 subsulcate and not bearing fovea(e) at or near middle of elytron; i8 with or without subhumeral fovea: if present, then fovea shallow and bearing seta or small, perforate, and located at basal fourth near seta Eo4; if i8 lacking posthumeral foveae, elytron with 8 entirely punctate interneurs. ABL = 1.84–3.04 mm. (Erwin 1974a; Erwin et al. 2002).

#### Distribution.

Species of this subgenus are restricted to the New World, described from México south to Argentina and some of the Caribbean islands.

## Discussion

Both species of the Meotachys subgenus Hylotachys described above are the first “bifoveate’ (Ortuño and Arillo 2015) tachyines discovered to possess mesepisternal pits. These structures are highly varied in form and are found in “non-bifoveate” species throughout Elaphropus (Tachyura) and allied subgenera (incl. *Tachylopha*, *Barytachys*, *Nototachys*, and *Sphaerotachys*) (Erwin 1970), as well as a small subgenus of South American *Bembidion* (Maddison 2014) and certain Oodini (Spence 1982). Nearly all species known to possess these structures are hygrophilous. The waxy substance noted by Maddison (2014) in ethanol-preserved specimens was not observed in any of the specimens of *Hylotachys* examined, which were likely not ethanol-killed.

Previously synonymized under *Polyderis* (Erwin 1974b) and later considered a subgenus, *Polyderidius* Jeannel 1962 should be considered a separate genus, united by consistent morphological characters and distinct from *Polyderis* s.str. Species of *Polyderidius* are instead probably allied with *Paratachys* and *Tachys* s.str., based on the form of the elytral apical recurrent groove.

*Nototachys* Alluaud, 1930 is a small but distinctive group whose name has been considered a subjective synonym of *Elaphropus* (Erwin 1974) or the Elaphropus subgenus Sphaerotachys (Sciaky and Vigna-Taglianti 2003), as a subgenus of *Elaphropus* (Bousquet 2012) or *Tachyura* (Kópecky 2003), or as a separate genus (Lorenz 2005). The newly described South American species, Elaphropus (Nototachys) occidentalis, expands the known distribution of this previously Old World-restricted subgenus. *Elaphropus occidentalis* shares aspects of its overall form with *Elaphropus comptus* (Andrewes 1922), the type species of *Nototachys*, and its aberrant discal elytral chaetotaxy with Elaphropus (Nototachys) comptus borealis (Andrewes 1925).

Relationships among groups within the subtribe Tachyina, in particular *Elaphropus*, remain a subject of contention and have been reviewed by several authors in recent decades. The conflicting taxonomic concepts proposed in previous reviews, classifications, and checklists (Lindroth 1966, Erwin 1974b, Shilenkov 2002, Kopecký 2003, Sciaky and Vigna-Taglianti 2003, Lorenz 2005, Bousquet 2012) represent alternative hypotheses waiting to be tested in a molecular context.

Due to their small size and a lack of resources for their identification, tachyines are easy to overlook or misidentify. A good deal of undescribed tachyine diversity is likely hidden in uncurated material, stored bycatch, and existing collections (Baehr 2016). In the Amazon Basin, ecosystem level processes are thought to have generated the rapid diversification apparent in this and other groups (Erwin and Adis 1978). Detailed collection data and large sample sizes exist for a number of New World species discovered through long term, bulk collecting efforts employing passive traps, leaf litter sampling, and canopy fogging (Erwin 1983, 1984, 1991). Much less is understood of the way of life, intraspecific diversity, and distribution of species described from small series and with limited representation in collections. Conservation status is difficult to estimate for such rarely collected taxa as *Costitachys* and *Tachyxysta*, especially for those known only from unique collecting events (e.g., *Stigmatachys* and Elaphropus (Idiotachys)). Moreover, anthropogenic impact to rapidly developing regions such as the Atlantic coast of South America has likely already affected the distribution and abundance of both described and undiscovered tachyine species.

## Supplementary Material

XML Treatment for Moirainpa

XML Treatment for Micratopus

XML Treatment for Lymnastis

XML Treatment for Costitachys

XML Treatment for Tachyta

XML Treatment for Tachyxysta

XML Treatment for Tachyxysta howdenorum

XML Treatment for Elaphropus

XML Treatment for Tachyura

XML Treatment for Barytachys

XML Treatment for Ammotachys

XML Treatment for Elaphropus (Ammotachys) marchantarius

XML Treatment for Idiotachys

XML Treatment for Elaphropus (Idiotachys) acutifrons

XML Treatment for Nototachys

XML Treatment for Elaphropus (Nototachys) occidentalis

XML Treatment for Porotachys

XML Treatment for Polyderis

XML Treatment for Liotachys

XML Treatment for Tachysbembix

XML Treatment for Tachys

XML Treatment for Paratachys

XML Treatment for Polyderidius

XML Treatment for Stigmatachys

XML Treatment for Stigmatachys uvea

XML Treatment for Nothoderis

XML Treatment for Meotachys

XML Treatment for Scolistichus

XML Treatment for Meotachys (Scolistichus) riparius

XML Treatment for Hylotachys

XML Treatment for Meotachys (Hylotachys) ballorum

XML Treatment for Meotachys (Hylotachys) rubrum

XML Treatment for Pericompsus

XML Treatment for Pericompsus sensu stricto

XML Treatment for Eidocompsus
